# Clinical, pathological, and genetic characteristics of 23 DMD patients in northern China

**DOI:** 10.1186/s13023-026-04488-y

**Published:** 2026-07-13

**Authors:** Yi Bu, Jingzhe Han, Jinliang Deng, Pingping Fang, Di An, Guang Ji, Shaojuan Ma, Xueqin Song

**Affiliations:** 1 Department of Neurology, The Second Hospital of Hebei Medical University, Shijiazhuang, Hebei 050000 China; 2 Department of Neurology, Affiliated Hospital of Chengde Medical College, Chengde, Hebei 067000 China; 3 Department of Neurology, Harrison International Peace Hospital, Hengshui, Hebei 053000 China; 4 Department of Neurology, Handan Central Hospital, Handan, Hebei 056000 China; 5 Department of Neurology, Affiliated Hospital of Hebei University, Baoding, Hebei 071000 China; 6 Key Laboratory of Clinical Neurology, Ministry of Education, Hebei Medical University, Shijiazhuang, Hebei 050000 China; 7 Key Neurological Laboratory of Hebei Province, Shijiazhuang, Hebei 050000 China

**Keywords:** Duchenne muscular dystrophy, Clinical, Pathological, Genetic characteristics, Correlation analysis

## Abstract

**Background:**

Duchenne muscular dystrophy (DMD) is a rare X-linked neuromuscular disorder characterised by heterogeneous early manifestations. This study summarised the clinical, pathological, and genetic characteristics of patients with DMD in northern China and identified useful indicators for diagnosis and disease assessment.

**Methods:**

Twenty-three DMD patients at the Second Hospital of Hebei Medical University between 2014 and 2022 were retrospectively reviewed. Clinical data, laboratory findings, electrocardiography, electromyography, muscle pathology, dystrophin immunohistochemistry, and DMD gene variants were analysed.

**Results:**

Age at onset ranged from 0.5 to 7.5 years, mainly between 3.5 and 6.0 years. Bilateral lower-limb weakness and exercise intolerance were observed in 21 patients. All patients had elevated myocardial enzymes, decreased serum creatinine, elevated inorganic phosphate, and myogenic changes on electromyography; 22 had elevated transaminases and 17 had abnormal electrocardiograms. Muscle pathology showed connective tissue and fatty infiltration in all cases, with fibre atrophy. Dystrophin immunohistochemistry revealed complete loss of dystrophin-N/C/R in 21 patients and partial loss in two patients. Muscle strength was positively correlated with CK, CKMB, ALT, AST, LDH, and creatinine. Genetic testing identified 15 deletions, five duplications, and three small mutations, including a novel frameshift variant. Among 21 patients with parental testing, 10 mothers were carriers and 11 cases were considered de novo.

**Conclusions:**

DMD exhibited distinct clinicopathological and genetic patterns. Unexplained transaminase or myocardial enzyme elevation, decreased serum creatinine, and early motor abnormalities should prompt DMD genetic testing.

## Introduction

Duchenne muscular dystrophy (DMD) is a rare X-linked recessive muscle-wasting disorder caused by mutations in the DMD gene on the X chromosome, resulting in loss of dystrophin in the skeletal muscle cell membrane. This condition primarily affects males [1]. Variants in the DMD gene, including complete or partial defects, lead to qualitative and quantitative abnormalities of dystrophin expression, resulting in conditions such as DMD, Becker muscular dystrophy (BMD), and X-linked dilated cardiomyopathy (XLDCM) [2]. Out-of-frame mutations in the DMD gene typically result in complete or near-complete loss of dystrophin, leading to the severe phenotype observed in DMD. In contrast, in-frame mutations often produce truncated dystrophin and are associated with the milder phenotype of BMD [1]. Although patients with DMD are born with relatively normal muscle strength, motor function deteriorates progressively with age. Typically, boys lose the ability to walk before the age of 12, eventually becoming completely paralyzed and experiencing progressive respiratory decline. Most patients have a life expectancy of approximately 25 years [3]. Early symptoms of DMD can be subtle and may be overlooked by parents or clinicians, often resulting in misdiagnosis and delayed diagnosis. Due to the nonspecific nature of clinical manifestations and limited awareness among healthcare providers, DMD is often misdiagnosed as other conditions such as polymyositis or progressive muscular atrophy. This study aimed to summarise the clinical, pathological, and genetic characteristics of patients with DMD at our centre to enhance clinicians’ awareness of the disease and provide a theoretical basis for its diagnosis.

## Methods

### Subjects investigated

A total of 23 patients diagnosed with Duchenne muscular dystrophy (DMD) through muscle pathology and genetic testing were enrolled at the Second Hospital of Hebei Medical University between January 2014 and December 2022. Clinical data were collected, including gender, age, initial symptoms, limb muscle strength, laboratory test results, electrocardiograms, electromyography findings, and genetic test results. Other neuromuscular diseases, such as myasthenia gravis and spinal muscular atrophy, were excluded based on definitive diagnostic criteria.

This study was approved by the Medical Ethics Committee of the hospital (2022-P005), and informed consent was obtained from the legal guardians of all participants.

### Collection and processing of muscle specimens

One patient underwent descending muscle biopsy under general anesthesia, while the remaining patients underwent descending open biceps or quadriceps muscle biopsy under local anesthesia. Excised muscle samples were immediately fixed in isopentane precooled with liquid nitrogen and then sectioned into 8-micron-thick slices using a frozen microtome. Muscle sections from all patients were stained with hematoxylin-eosin (HE), modified Gomori trichrome (MGT), oil red O (ORO), succinate dehydrogenase (SDH), nicotinamide adenine dinucleotide tetrazolium reductase (NADH-TR), ATP enzyme (pH 4.5, pH 10.2), nerve-specific enolase (NSE), and periodic acid-Schiff (PAS). All patients also underwent immunohistochemical staining using dystrophin antibodies: dystrophin-C, cystrophin-N, and dystrophin-R. The remaining muscle tissue was stored at -80 °C. Pathological features of each patient were documented in detail.

### Genetic testing

Blood samples (2 mL) were collected from all patients and consenting family members for genetic testing. After anticoagulation with ethylenediaminetetraacetic acid (EDTA), DNA was extracted using a blood DNA extraction kit (Beijing Total Gold Biotechnology Co., Ltd.). The extracted DNA was analyzed using exome sequencing. If deletions or duplications were identified, multiplex ligation-dependent probe amplification (MLPA) was used for confirmation. For single nucleotide variants, Sanger sequencing was performed.

## Statistical methods

Statistical analysis was performed using GraphPad Prism 9. Data are expressed as mean ± standard deviation (X ± SD). Categorical data are presented as frequencies and percentages.

## Results

### Demographic characteristics

**Fig. 1:**
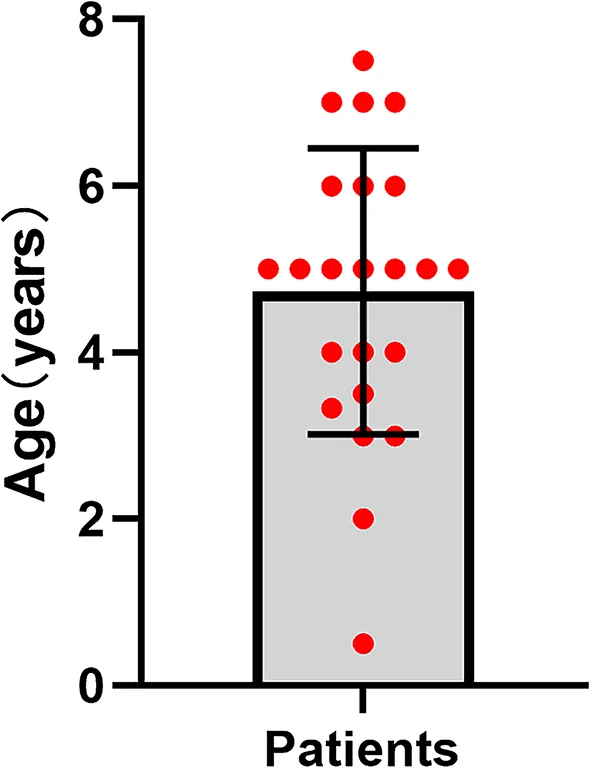
Distribution of age of onset in DMD patients

All 23 patients were male, with age at onset ranging from 0.5 to 7.5 years. The majority of patients (15/23, 65.2%) had onset between 3.5 and 6 years of age (Fig. 1). Symptom onset was insidious in all patients, and disease progression followed a cyclical course lasting from 4 months to 5 years (Table 1). Of the 23 patients, 2 (2/23, 8.7%) had a family history, whereas 21 (21/23, 91.3%) were sporadic cases.

**Table 1:** Demographic characteristics, reasons for presentation, clinical manifestations, symptoms, and laboratory findings in dmd patients

Factor	Patients (*n* = 23)	Mean ± SD
**Demographic characteristics**		
Sex	Male(100%)	
Age of treatment	2.33–15.58	
Family history	2/23(8.70%)	
Chronic disease	23/23(100%)	
Disease course	4 m-7years	
**The reason for the visit**		
Fever	1/23(4.35%)	
Physical examination found increased muscle enzymes	2/23(8.70%)	
Physical examination revealed abnormal liver function	2/23(8.70%)	
**Clinical manifestation**		
Weak lower limbs	21/23(91.30%)	
Exercise intolerance	21/23(91.30%)	
Myodynia	2/23(8.70%)	
Mental decline	1/23(4.35%)	
Motor stunting	7/23(30.43%)	
ADHD	1/23(4.35%)	
Damaged myocardial performance	0	
Breathing hard	0	
**Physical examination**		
Ppositive Gowers Sign	17/23(73.91%)	
Hypertrophic gastrocnemius muscle	18/23(78.26%)	
The symmetrical muscle strength was decreased	20/23(86.96%)	
Asymmetric muscle strength decreases	3/23(13.04%)	
Decreased muscle strength in both lower limbs	21/23(91.30%)	
Decreased muscle strength in both upper limbs	15/23(65.21%)	
Decreased muscle strength in proximal muscle	21/23(91.30%)	
Decreased muscle strength in distal muscle	18/23(78.26%)	
**Electrocardiogram**		
Normal electrocardiogram	6/23(26.09%)	
Abnormal electrocardiogram	17/23(73.91%)	
Sinus irregularity	10/17(58.82%)	
High voltage in the left ventricle	4/17(23.53%)	
ST segment elevation	2/17(11.76%)	
qRs wave	1/17(5.88%)	
**Laboratory examination**		
Elevated CK in serum (U/L)	23/23(100%)	12476.8 ± 8798.2
Elevated CK-MB in serum (U/L)	23/23(100%)	420.64 ± 418.64
Elevated LDH in serum (U/L)	23/23(100%)	1026.84 ± 493.16
Elevated MYO in serum (U/L)	23/23(100%)	555.27 ± 280.24
Elevated ALT in serum (U/L)	22/23(95.65%)	263.29 ± 155.05
Elevated AST in serum (U/L)	22/23(95.65%)	189.61 ± 99.09
Decreased creatinine in serum (µmol/L)	23/23(100%)	20.36 ± 5.7
Elevated inorganic phosphate in serum(mmol/L)	23/23(100%)	1.89 ± 0.14
**Electrophysiology**		
Myogenic damage	23/23(100%)	
Myogenic impairment in both lower limbs	23/23(100%)	
Myogenic damage in both upper limbs	21/23(91.30%)	
Decreased motor unit action potential wave amplitude	23/23(100%)	
Fibrillation potential	8/23(34.78%)	
Positive sharp wave	23/23(100%)	
**Immunohistochemical staining**		
Dystrophin-N\C\R completely missing	21/23(91.30%)	
Dystrophin-N\C\R most of the missing	2/23(8.70%)	

### Reasons for presentation and clinical presentation

The reasons for presentation varied. One patient (1/23, 4.35%) initially presented with fever, 2 patients (2/23, 8.70%) had abnormal liver function (elevated transaminase levels), 2 patients (2/23, 8.70%) had elevated muscle enzymes (creatine kinase, creatine kinase isoenzymes), and 2 patients (2/23, 8.70%) reported muscle pain. Other presentations included 1 patient (1/23, 4.35%) with reduced motor function, 7 patients (7/23, 30.43%) with growth retardation, and 1 patient (1/23, 4.35%) with attention deficit hyperactivity disorder (ADHD).

The most prominent clinical manifestation was bilateral lower limb weakness and exercise intolerance, which were present in 21 patients (21/23, 91.3%). On physical examination, 20 patients (20/23, 86.96%) showed symmetrical muscle strength reduction, 21 patients (21/23, 91.3%) had decreased proximal muscle strength, and 18 patients (18/23, 78.26%) had reduced distal muscle strength. In addition, 18 patients (18/23, 78.26%) exhibited gastrocnemius pseudohypertrophy, and 18 patients had a positive Gowers’ sign (Table 1).

### Laboratory examination

#### Serum myocardial enzymes

All 23 patients had elevated myocardial enzymes. The mean serum creatine kinase (CK) was 12,476.8 ± 8,798.2 U/L (reference range: 50–310 U/L), CK-MB was 420.64 ± 418.64 U/L (reference range: 0–24 U/L), lactate dehydrogenase (LDH) was 1,026.84 ± 493.16 U/L (reference range: 125–250 U/L), and myoglobin (MYO) was 546.58 ± 280.24 ng/mL (reference range: 0–85 ng/mL) (Table 1).

#### Electrocardiogram (ECG)

Of the 23 patients, 6 (6/23, 26.09%) had normal ECG findings, whereas 17 (17/23, 73.91%) had abnormal ECG findings. Among patients with normal ECG, 2 (2/6, 33.33%) had repeat mutations, and 4 (4/6, 66.67%) had deletion mutations. Among patients with abnormal ECG, 3 (3/17, 17.65%) had repeat mutations, 11 (11/17, 64.71%) had deletions, and 3 (3/17, 17.65%) had minor mutations. Abnormal ECG findings included sinus arrhythmia (10/17, 58.82%), left ventricular high voltage (4/17, 23.53%), ST segment elevation (2/17, 11.76%), and QRS wave abnormalities (1/17, 5.88%). For repeat mutations, 40% (2/5) of patients with normal ECG had mutations in exons 3–5 and 44–55, while 60% (3/5) with abnormal ECG had mutations in exons 2, 5–6, and 46–59. Among deletion mutations, 26.67% (4/15) of patients with normal ECG had deletions in exons 3–5 and 44–55, while 73.33% (11/15) with abnormal ECG had deletions in exons 2, 5–6, and 46–59.

#### Electromyogram (EMG)

All 23 patients (23/23, 100%) showed myogenic damage. Typical findings included reduced action potential amplitude, shortened duration, and increased polyphasic waves. Fibrillation potentials were observed in 8 patients (8/23, 34.78%), and 2 patients (2/23, 8.7%) showed no abnormalities in both upper limbs.

#### Liver function

Twenty-two patients (22/23, 95.65%) had elevated transaminases (ALT: mean 263.29 ± 155.05 U/L, reference range: 9–50 U/L; AST: mean 189.61 ± 99.09 U/L, reference range: 15–40 U/L), and viral hepatitis and drug-induced liver injury were excluded in all cases (Table 1).

#### Renal function and electrolytes

All 23 patients (23/23, 100%) showed abnormal renal function, with decreased serum creatinine (mean 20.36 ± 5.1 µmol/L, reference range: 57–97 µmol/L) and elevated inorganic phosphate (mean 1.89 ± 0.14 mmol/L, reference range: 0.85–1.51 mmol/L) (Table 1).

### Histopathological findings

#### Pathological staining

**Fig. 2:**
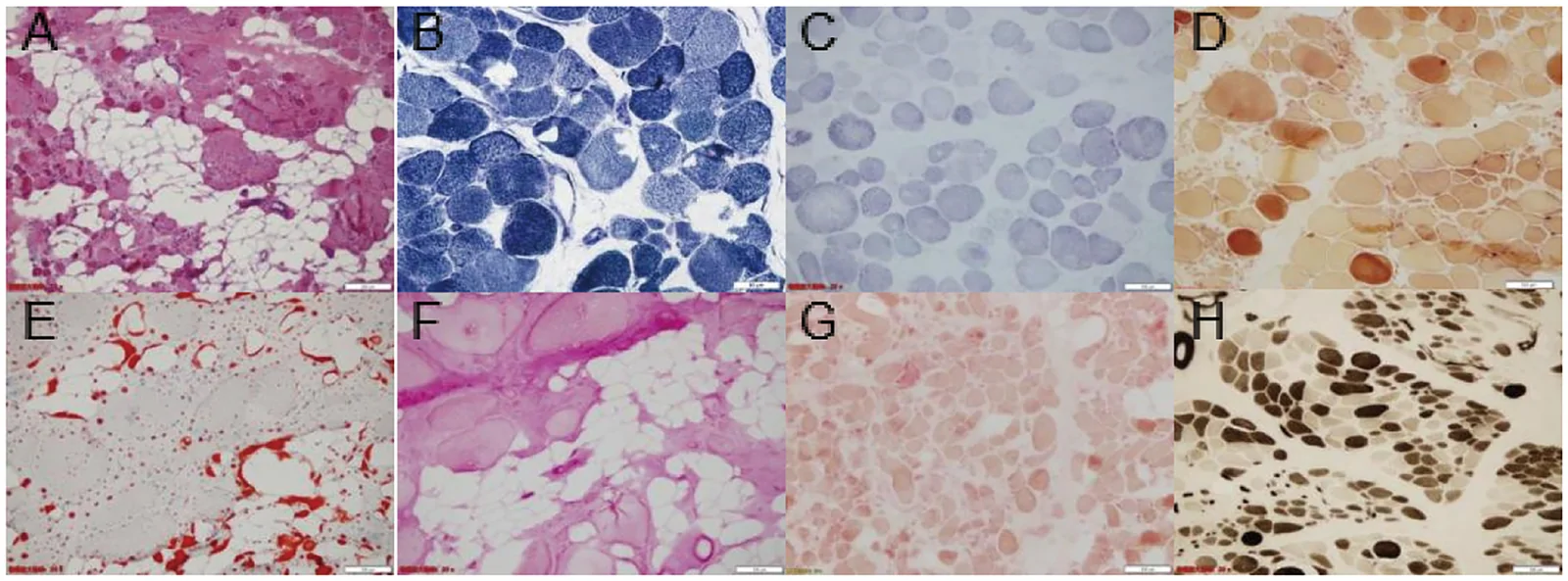
Histopathological features of skeletal muscle in DMD patients. (**A**) HE staining revealed uneven muscle fiber sizes (200); (**B**) NADH staining demonstrated uneven distribution of oxidases in muscle fibers, with some fibers showing “insect etching”-like alterations (200×); (**C**) SDH staining showed uneven oxidase distribution, with some muscle fibers deeply stained or exhibiting deep staining at the edges (200); (**D**) NSE staining revealed some muscle fibers with deep staining (200×); (**E**) Oil Red O staining showed significantly increased lipid droplets in the muscle fibers (200); (**F**) PAS staining indicated increased glycogen content in the muscle fibers (200×); (**G**) ACP staining showed increased enzyme activity in some areas (200×); (**H**) ATP enzyme staining demonstrated a mosaic distribution of muscle fibers, with atrophy affecting both fiber types (pH = 10.2, 200×).(scale bars, 100 μm)

HE staining showed indistinct myofiber bundle borders in 10 patients (10/23, 43.48%). All patients exhibited connective tissue and fatty infiltration, without inflammatory cell infiltration in muscle fibers (Fig. 2A). NADH-SDH-NSE staining revealed an uneven distribution of oxidative enzymes in 20 patients (20/23, 86.96%), with some muscle fibers showing intense staining. Two patients (2/23, 8.7%) showed no abnormalities, and one patient (1/23, 4.35%) exhibited insect-like changes (Fig. 2B, C and D). ORO and PAS staining indicated mild increases in lipid droplets and glycogen in 14 patients (14/23, 60.87%), marked increases in 3 patients (3/23, 13.04%) and no abnormalities in 6 patients (6/23, 26.09%) (Fig. 2E and F). ACP enzyme staining showed mild abnormalities in 17 patients (17/23, 73.91%) and no abnormalities in 6 patients (6/23, 26.09%) (Fig. 2G). ATP enzyme staining demonstrated both type I and type II muscle fiber atrophy in all 23 patients (100%) (Fig. 2H).

#### Immunohistochemical staining

**Fig. 3:**
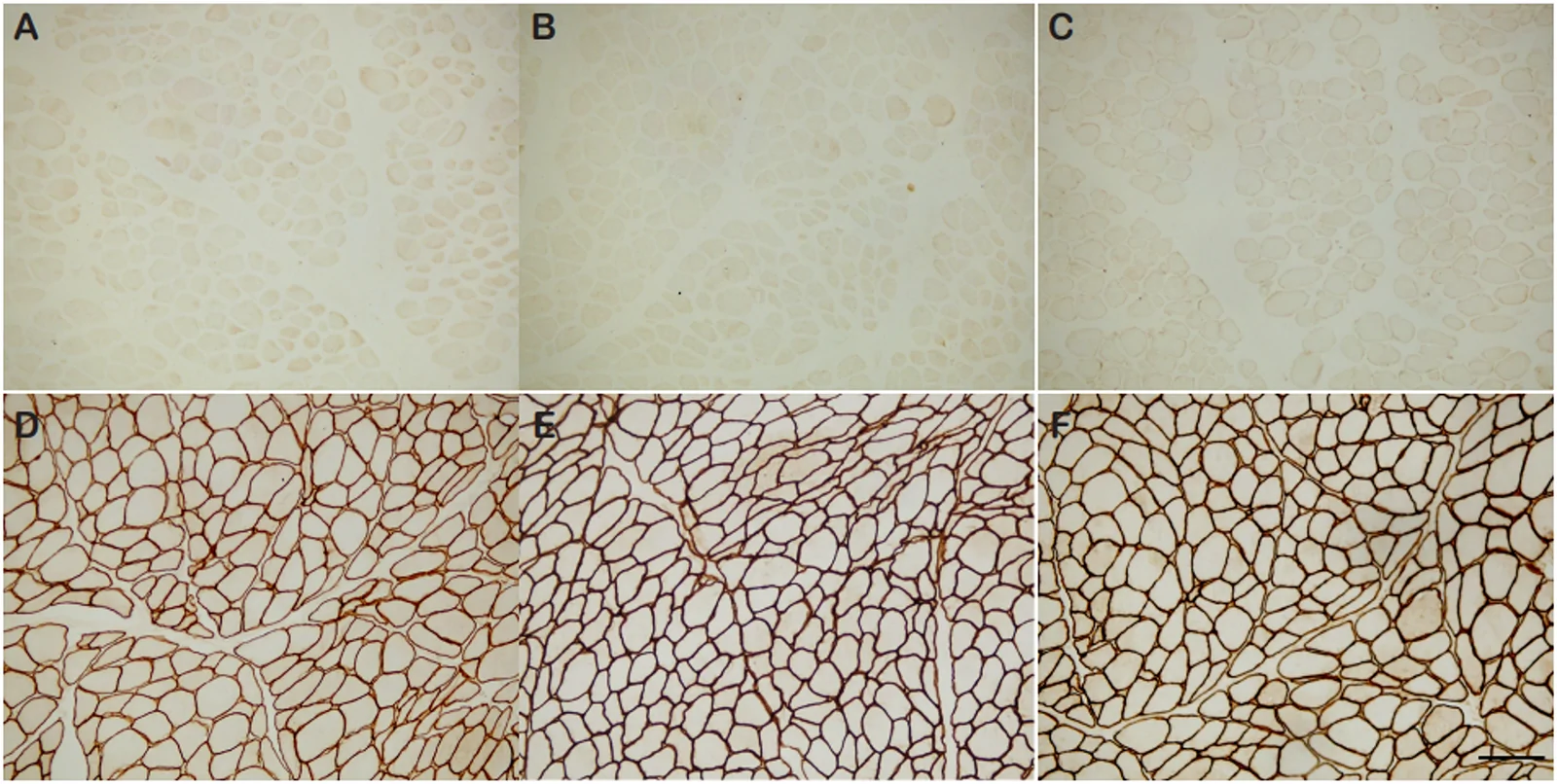
Characteristics of immunohistochemical staining of skeletal muscle in DMD patients. (**A**) No expression of dystrophin Dystrophin-N on myofiber cell membranes in DMD patients compared to the control (200×); (**B**) No expression of dystrophin Dystrophin-C on the myofiber cell membrane in DMD patients compared to the control (200×); (**C**) No expression of dystrophin Dystrophin-R on the myofiber cell membrane in DMD patients compared to the control (200×).(scale bars, 100 μm)

Immunohistochemical staining revealed complete loss of dystrophin-N, dystrophin-C, and dystrophin-R expression in the muscle fiber membrane in 21 patients (21/23, 91.3%) (Fig. 3A-C). Two patients (2/23, 8.7%) showed partial loss of dystrophin. One patient (Case 11) showed almost complete absence of dystrophin-C and dystrophin-N, and the other patient (Case 13) showed nearly complete loss of dystrophin-C and dystrophin-N with partial loss of dystrophin-R.

### Correlation analysis

**Fig. 4:**
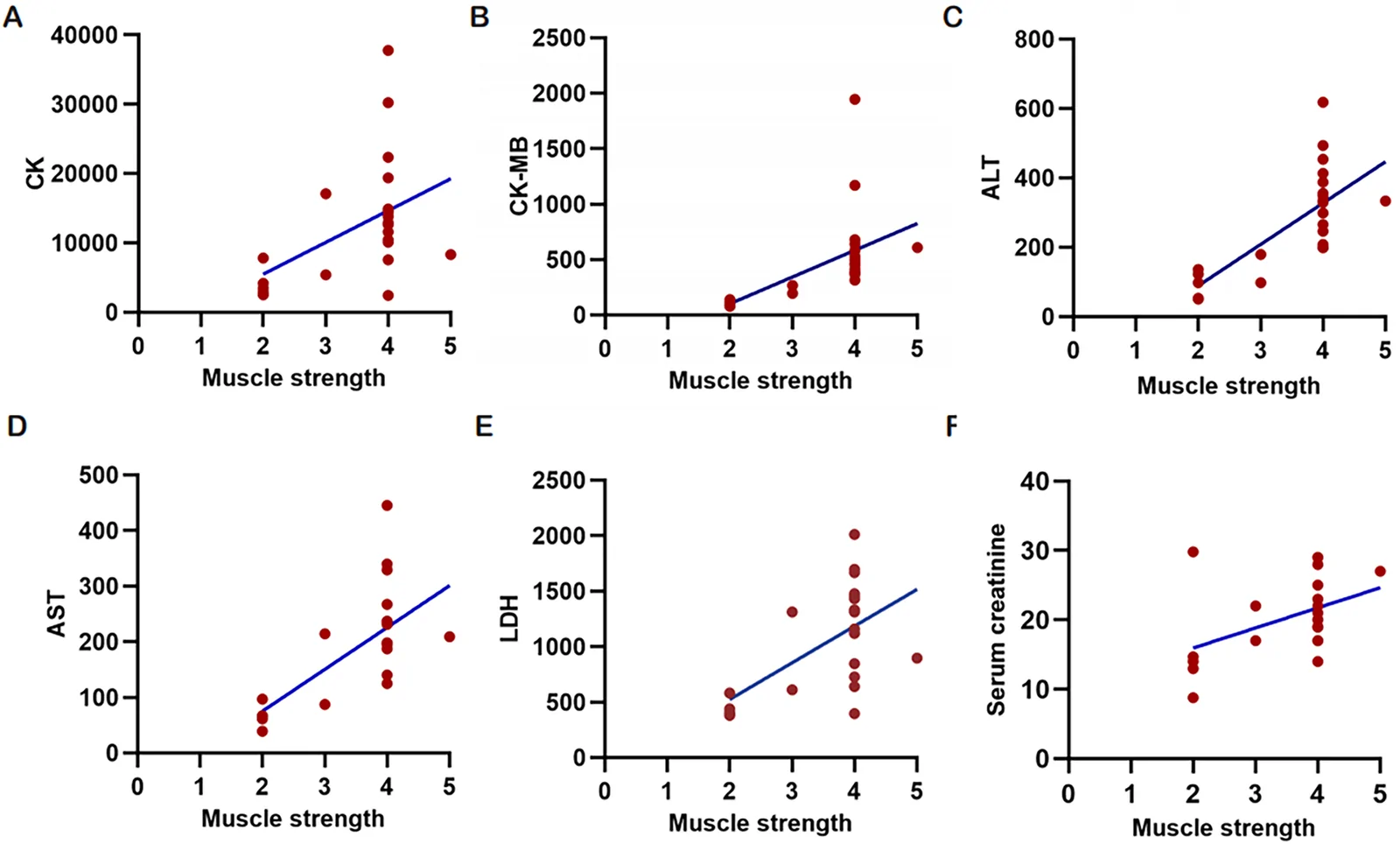
Correlation analysis between laboratory-related indicators and muscle strength in DMD patients. **A**. Muscle strength was positively correlated with CK; **B**. Muscle strength was positively correlated with CK-MB; **C**. Muscle strength was positively correlated with ALT; **D**. Muscle strength was positively correlated with AST; **E**. Muscle strength was positively correlated with LDH; **F**. Muscle strength was positively correlated with Serum creatinine

Correlation analysis of the laboratory indices with muscle strength showed that as muscle strength decreased, serum levels of CK, CK-MB, ALT, AST, LDH, and creatinine also decreased. Muscle strength was positively correlated with these laboratory indices (Fig. 4A-F).

### Genetic results

**Fig. 5:**
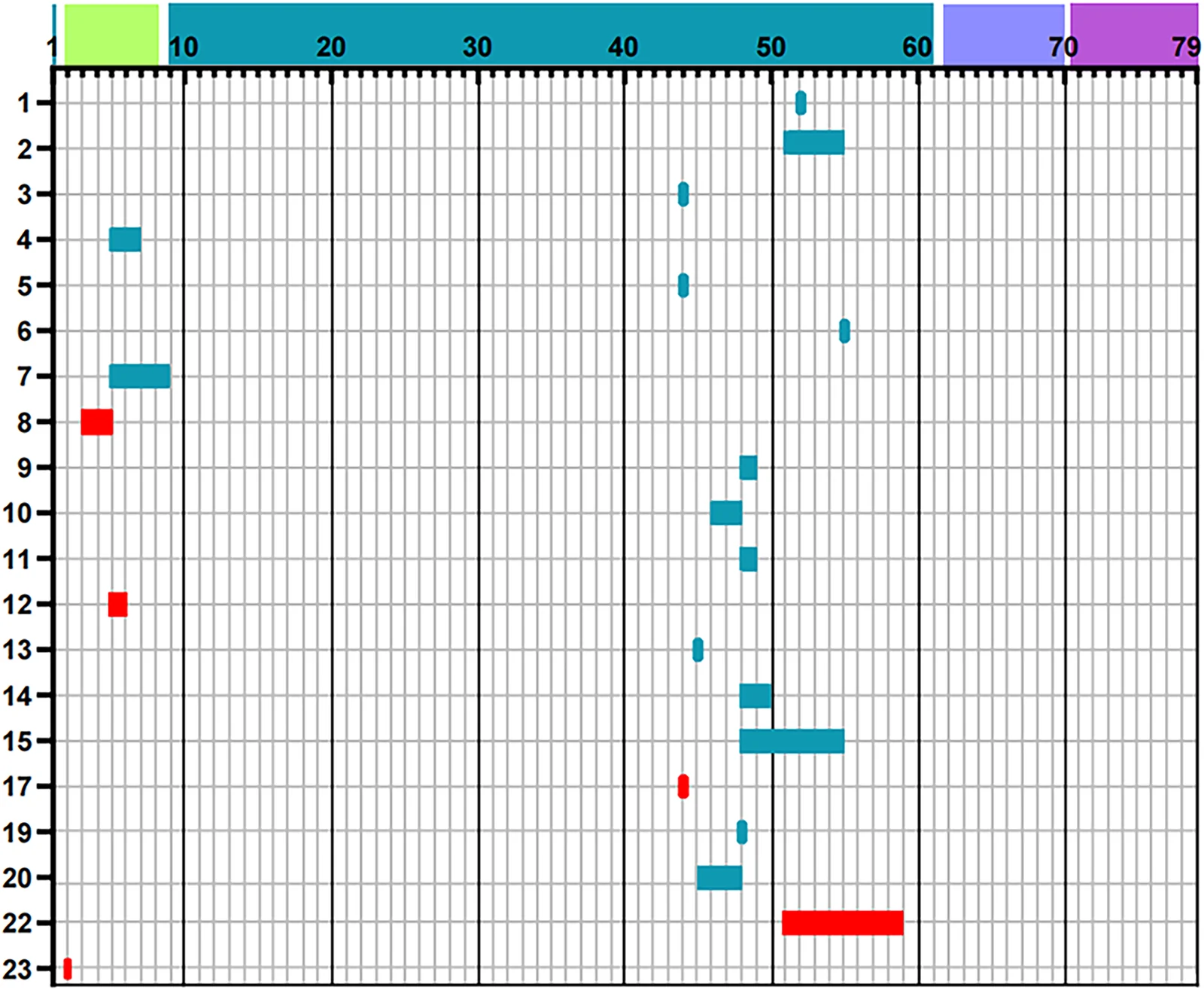
Map of gene deletion and duplication in DMD patients. (Note: Blue is DMD gene deletion mutation, red is repeat mutation)

Among the 23 patients, 15 (15/23, 65.22%) had DMD gene deletions, including 10 with large-segment deletions. Five patients (5/23, 21.74%) had duplications, including 2 with large-segment duplications (Fig. 5). Three patients (3/23, 13.04%) had minor mutations, including two missense mutations and one frameshift mutation (a new pathogenic mutation)(Table 2). Parental testing was conducted in 21 of the 23 patients, and 10 mothers (10/23, 47.62%) were identified as carriers with the same mutation as their affected children. The remaining 11 patients (11/23, 52.38%) had de novo mutations.

**Table 2:** Information on minor mutations in DMD patients

Number	Nucleotide change	Amino acid changes	Exon	variation	ACMG grade	Mother carries	Is reported
16	c.5938delG	p.E1980fs*3	42	frame shift	Likely pathogenic	Yes	No
18	c.512 C > T	p.A171V	6	missense	Likely pathogenic	Yes	Yes
21	c.9337 C > T	p.R3113X	64	missense	Pathogenic	No	Yes

### Treatment and prognosis

Fifteen patients were treated with corticosteroids (0.75 mg/kg/day for at least 3 months). Follow-up durations ranged from 6 months to 3 years, during which changes in exercise capacity, muscle enzymes, liver function, and glucocorticoid treatment were assessed. Among these patients, 3 discontinued medication due to side effects (2 developed obesity and 1 developed impaired glucose tolerance), while the remaining 12 continued low-dose prednisone acetate. Among the 12 patients, 7 showed no significant progression after treatment, 3 showed a reduction in muscle enzymes after 2 months of hormone therapy, and 2 showed slow disease progression. Among the 8 patients who did not receive hormone therapy, 6 showed disease progression and 2 showed no significant progression. Glucocorticoid-related adverse reactions in this cohort were mainly metabolic abnormalities, without severe hepatic or skeletal system adverse events. Short-term hormone therapy was effective in slowing disease progression.

## Discussion

DMD is a monogenic disorder caused by mutations in the DMD gene, which encodes dystrophin. Because the DMD gene is located on the X chromosome, inheritance follows an X-linked recessive pattern; males with the mutation are affected, whereas females are typically carriers [4]. In this study, patient ages ranged from 2.3 to 15.6 years, with symptom onset occurring between 0.5 and 7.5 years, most commonly between 3.5 and 6 years. This is consistent with previous reports indicating that most patients with DMD experience onset between 3 and 5 years of age.

The onset of DMD is often insidious, with early clinical manifestations that are difficult to detect. Common early features include delayed motor milestones, such as difficulty running, frequent falls, and a waddling gait. These initial symptoms are easily overlooked by both parents and healthcare providers. In this study, two children initially sought medical attention due to abnormal liver function identified during routine examinations, highlighting the atypical early presentation of DMD. This also supports previous reports that abnormal liver function may represent an early clinical manifestation of DMD [5]. Regarding the mechanism of liver dysfunction in DMD, transaminases are present not only in hepatocytes but also in skeletal muscle, cardiac muscle, red blood cells, renal tubular cells, and other tissues. Therefore, skeletal muscle damage can lead to elevated transaminase levels, potentially mimicking liver injury. This underscores the importance of considering muscle injury, particularly DMD, when unexplained elevated transaminases are detected in pediatric patients. In this study, two patients presented with elevated myocardial enzymes as their initial clinical sign, without early limb weakness. This finding highlights the clinical heterogeneity of DMD, in which myocardial enzyme elevation may precede skeletal muscle weakness. Such cases further emphasize the need to consider DMD in patients with isolated elevation of myocardial enzymes.

All patients in this study exhibited lower limb weakness and exercise intolerance, with most showing reduced proximal muscle strength. Six patients were unable to stand due to severe muscle weakness. Gastrocnemius hypertrophy was observed in the majority of patients, suggesting replacement of muscle fibers by connective tissue and fat, consistent with previous findings [6]. One patient presented with asymmetric muscle weakness at the age of 1, which may be related to individual variation and early disease onset. Another patient had ADHD, and one experienced cognitive decline. Increasing evidence suggests that patients with DMD are at high risk of neurodevelopmental comorbidities, including intellectual disability, autism spectrum disorder (ASD), ADHD, and other cognitive disorders. In some cases, these neurological manifestations may represent the first clinical signs of DMD [7]. This is attributed to the presence of seven dystrophin isoforms encoded by the DMD gene, which are widely expressed in the central nervous system. Mutations in the DMD gene disrupt the function of these isoforms, leading to cognitive and developmental impairments. The Dp140 isoform, primarily expressed during fetal and early childhood development, is closely associated with cognitive function. Loss or dysfunction of Dp140 can result in learning disabilities and intellectual impairment. In addition, Dp71, which is widely expressed in brain neurons, is involved in synaptic plasticity and neural signaling. Abnormalities in Dp71 may lead to neurotransmitter imbalance, contributing to ADHD symptoms. Therefore, when patients present with both muscle weakness and neurodevelopmental abnormalities, clinicians should consider the possibility of DMD to avoid misdiagnosis and ensure early intervention.

Due to mutations in the DMD gene, the integrity of muscle fibers is compromised, resulting in muscle damage during physical activity. This leads to the rapid release of serum creatine kinase, creatine kinase isoenzymes, and phosphates into the bloodstream, causing significant elevations in serum creatine kinase levels and other related indicators. All patients in this study exhibited markedly elevated creatine kinase levels, with most showing severe increases, likely reflecting disruption of muscle fiber integrity. Mild elevation of creatine kinase in some patients may be attributed to reduced physical activity due to advanced muscle weakness in later disease stages, as well as a decreased number of residual muscle fibers. Additionally, elevated glutamate transaminase levels were observed in most patients. As previously noted, transaminases are present in skeletal muscle cells, and their elevation may reflect muscle injury. Serum myocardial enzymes and transaminases serve as markers of the extent of muscle damage. To further explore the relationship between transaminase and myocardial enzyme levels and limb weakness, a correlation analysis was conducted. The results indicated that lower transaminase and myocardial enzyme levels were associated with greater limb weakness. This finding, which has not been extensively reported previously, provides additional insight for clinical practice and may allow a simpler assessment of DMD severity. In confirmed DMD patients, lower transaminase levels are associated with more severe muscle damage. In this study, a subset of patients exhibited low serum creatinine levels, consistent with previous reports [8]. Furthermore, it has been reported that DMD patients have significantly lower serum creatinine levels compared with BMD patients, and that creatinine levels correlate with disease severity in DMD [9]. Our findings showed that as muscle strength decreased, serum creatinine levels also declined, with a positive correlation between the two. The correlation between muscle strength and laboratory indicators provides a theoretical basis for clinical evaluation. However, other laboratory indicators, such as inorganic phosphate levels, did not show a significant correlation with muscle strength, which may be due to the limited sample size in this study.

The deletion of the dystrophin gene results in an unstable cardiomyocyte membrane, which becomes more susceptible to damage under mechanical stress, leading to cardiomyocyte death and fibrosis [10]. Our findings indicated that the severity of ECG abnormalities in DMD patients worsened with age, consistent with previous research [11]. Notably, in Case 22, despite a muscle strength grade of 2, the ECG findings were normal. This discrepancy may be attributed to the strong compensatory capacity of the heart, as cardiac function may remain preserved in the early stages of cardiomyocyte injury, resulting in no obvious ECG changes. Moreover, Case 22, who had elevated muscle enzymes and intermittently used coenzyme Q10, may have experienced delayed progression of ECG abnormalities due to medication effects. These findings suggest that clinicians should perform ECG examinations in patients with limb weakness and elevated muscle enzymes, as they may reveal early cardiac involvement. Even when ECG findings are normal, further cardiac evaluations, such as echocardiography or cardiac magnetic resonance imaging, may be necessary for comprehensive assessment.

Due to the irregular size of muscle fibers and instability of the muscle cell membrane, EMG in patients with DMD often reveals myogenic damage. In this study, some patients exhibited abnormalities confined to the proximal muscles of both lower limbs, while upper limb muscles showed no abnormalities. This discrepancy may be explained by the early stage of disease, in which the upper limbs are relatively spared. The larger lower limb muscles are subjected to greater mechanical load, resulting in more pronounced muscle fiber damage. In addition, EMG is more sensitive to abnormalities in lower limb muscles, which may account for the absence of early detectable changes in the upper limbs [6]. Notably, 8 patients exhibited fibrillation potentials on EMG, which are primarily associated with muscle fiber degeneration and regeneration, leading to local neuromuscular dysfunction and transient denervation of some muscle fibers. Therefore, when clinicians encounter patients with limb weakness, elevated muscle enzymes, and EMG evidence of myogenic damage, the presence of fibrillation potentials should be interpreted in conjunction with clinical manifestations and pathological findings.

Histopathological findings in this study demonstrated extensive muscle fiber degeneration and necrosis, with some fibers showing fatty infiltration, consistent with previous studies [12]. Interestingly, two patients in this study had relatively well-preserved muscle fibers, and immunohistochemical analysis revealed partial loss of dystrophin protein expression. While most patients with DMD exhibit complete loss of dystrophin protein (N, C, and R domains), some retain partial expression of specific dystrophin isoforms, particularly the R domain [13]. In these two patients, who harbored missense and duplication mutations, no dystrophin gene mutations were identified in their parents. This suggests that missense mutations may lead to unstable mRNA, which, although affecting dystrophin structure, may still allow partial protein expression through post-translational modifications or other compensatory mechanisms. One patient (Case 23) had a duplication mutation located in exon 2, which encodes the N-terminal region of dystrophin. This mutation may affect dystrophin expression and its interaction with actin, thereby impairing muscle fiber stability. Furthermore, the mothers of these patients did not carry detectable dystrophin gene mutations, suggesting that these cases may represent chimeric variants. Such variants may result in partial expression of normal or functional dystrophin in some muscle fibers, leading to relative preservation of dystrophin function [14].

Genetic testing is considered the gold standard for confirming Duchenne muscular dystrophy (DMD). The DMD gene, one of the largest human genes, spans approximately 2.5 Mb and contains 79 exons. Due to its large size, the gene is highly susceptible to mutations and exhibits diverse mutation types. These mutations are primarily classified into three categories: exon deletions, exon duplications, and small mutations [2]. MLPA (Multiplex Ligation-dependent Probe Amplification) technology can detect most deletions and duplications, whereas next-generation sequencing is effective for identifying point mutations [10]. In China, the most common mutations observed in patients with DMD include 54.3% deletions and 11.6% duplications, with deletion hotspots typically located in exons 45 to 54 [13]. In this study, MLPA and next-generation sequencing were used to analyze DMD genes in patients and their family members. Among the 23 patients, 56.7% had deletion mutations and 17.4% had duplication mutations. Deletion hotspots were identified in exons 5, 48, 49, 52, and 55, consistent with previous studies in China [15]. Interestingly, 11 mothers of the patients did not carry DMD gene mutations, suggesting that these mutations may be de novo. Several mechanisms may explain this finding: (1) the DMD gene, with its 79 exons and repetitive sequence structure, is prone to replication errors during oogenesis, increasing the likelihood of mutations; and (2) germline mosaicism, in which mutations occur in a subset of female germ cells, may also contribute. In such cases, some oocytes carry a mutated X chromosome, and the risk of affected male offspring depends on the proportion of mutant germ cells in the mother [2, 16]. Therefore, mothers of children with de novo mutations should be offered genetic counseling and prenatal consultation for future pregnancies. This study also identified a novel pathogenic mutation (Case 16), a frameshift mutation (c.5938 delG) in exon 42 of the DMD gene. This mutation was absent in the father and is not recorded in major genetic databases. According to ACMG guidelines [17], it is classified as “likely pathogenic”. Frameshift mutations in exon 42 are generally associated with more severe clinical phenotypes [18]. In this case, the patient was 11 years old with a 7-month disease course and showed rapid progression of limb weakness despite early diagnosis, consistent with the expected severity of frameshift mutations in the DMD gene.

Currently, there is no curative treatment for DMD, and the main therapeutic approaches include glucocorticoids and gene therapy. Glucocorticoids can reduce inflammation, slow muscle degeneration, and preserve muscle function. However, long-term use is associated with adverse effects such as weight gain, osteoporosis, and growth retardation. In this study, 15 patients received glucocorticoid therapy, and 3 discontinued treatment due to adverse effects. Nevertheless, short-term hormone therapy was associated with delayed disease progression compared with patients who did not receive hormone therapy, consistent with previous studies [19]. In recent years, novel glucocorticoid receptor modulators, such as vamorolone, have also been investigated, with the potential to maintain therapeutic efficacy while reducing the adverse effects associated with conventional corticosteroids [20]. Furthermore, gene therapy has made significant progress in the treatment of DMD. Current gene therapy approaches include exon skipping, gene replacement, gene editing, gene compensation, and cell therapy [21, 22]. Although these therapies remain under investigation, they offer promising potential for the treatment of DMD.

There are several limitations to this study: (1) this is a retrospective study, and some clinical data were incomplete, resulting in missing patient information; (2) the sample size is small, and further studies with larger cohorts are needed; (3) the study lacks long-term follow-up data, limiting further analysis of disease progression and longitudinal laboratory changes.

Clinical Take-Home Messages.

Isolated, unexplained elevations of transaminases or significant increases in muscle enzymes in children should not be simply diagnosed as primary liver disease; the possibility of DMD must be considered.

Serum CK, CKMB, ALT, AST, LDH and creatinine are positively correlated with muscle strength, which can be used as convenient laboratory markers for evaluating DMD disease severity and follow-up progression.

ECG abnormality rate is high in DMD patients; routine ECG screening is recommended at initial diagnosis, and further echocardiography or cardiac MRI is necessary even with normal ECG.

## Data Availability

All data generated or analysed during this study are available from the NCBI Sequence Read Archive under BioProject(PRJNA1279415).The novel variant c.5938 delG has been deposited in ClinVar (SCV006103423).

## Abbreviations

DMD: Duchenne Muscular Dystrophy
BMD: Becker Muscular Dystrophy
XLDCM: X-linked Dilated Cardiomyopathy
CK: Creatine Kinase
CKMB: Creatine Kinase-MB
LDH: Lactate Dehydrogenase
ALT: Alanine Aminotransferase
AST: Aspartate Aminotransferase
MYO: Myoglobin
ECG: Electrocardiogram
EMG: Electromyogram
HE: Hematoxylin-Eosin
MLPA: Multiplex Ligation-dependent Probe Amplification
NADH-TR: Nicotinamide Adenine Dinucleotide Tetrazolium Reductase
SDH: Succinate Dehydrogenase
